# Biomarkers of survival statistics (boss) analytical platform

**DOI:** 10.1186/2051-1426-3-S2-P99

**Published:** 2015-11-04

**Authors:** Michael McNamara, Ian Hilgart, William Redmond

**Affiliations:** 1 Earle A. Chiles Research Institute, Portland, OR, USA; 2 Providence Cancer Center, Portland, OR, USA; 3 Providence Health and Services, Portland, OR, USA

## Background

Immunotherapy is transforming how cancer is treated, and the Providence Portland Cancer Center has played an important role in this progress through clinical trials and pre-clinical research. However, even the most efficacious of these therapies only benefit a subset of patients and frequently cause significant immune-related adverse events (irAEs). Prognostic biomarkers that identify positively-responding patients, early in the course of therapy, are essential for guiding treatment decisions and improving patient outcomes. Evaluating how patients are responding to an immunotherapy regimen, and developing better treatment regimens, requires a detailed analysis of each patient's immune response to a given therapy.

Despite tremendous progress, robust prognostic biomarkers for clinical outcomes of cancer immunotherapy and overall survival (OS) remain elusive. The prognostic value of many putative biomarkers appears to erode as the patient population becomes more diverse (e.g. different cancer types, treatment regimens, etc). Furthermore, the individual data sets that are generated by the multiple analyses included in immunotherapy clinical trials can be enormous. The combination of size and complexity, along with the challenge of data normalization, limits the utility of the data. Identifying reliable immunological biomarkers for outcomes requires the ability to compare numerous parameters, across a diverse population of patients. Meeting this challenge requires specialized software tools to normalize, integrate and analyze these expansive data sets.

## Methods and results

Our research team has developed a software platform and series of algorithms that provide an innovative and powerful solution to this challenge. The software automates the process of normalizing and integrating data streams from disparate sources. The software can utilize data from conventional clinical lab assays (e.g. CBC, blood chem.), flow cytometry, transcriptomic, genomic, proteomic, array-based (chemokine/cytokine, antibody) and other data sets. Additionally, we have developed a visualization platform that allows our clinicians and scientists to access and interact with the data in a user-friendly setting. Most importantly, we have developed a novel algorithm to mine these integrated data sets for biomarkers that are correlated with clinical outcomes.

## Conclusions

This analytical platform has identified several putative biomarkers with prognostic utility for checkpoint blockade outcomes, and we are currently working to incorporate the data sets from additional trials being conducted at our institute.

Publicly accessible demonstrations of selected data visualizations from this project can be accessed at myCancerProject.org [http://www.mycancerproject.org].

